# Characterization and comparison of genomic profiles between primary cancer cell lines and parent atypical meningioma tumors

**DOI:** 10.1186/s12935-020-01438-x

**Published:** 2020-07-28

**Authors:** Eunhye Kim, Mirae Kim, Kyungha So, Young Seok Park, Chang Gok Woo, Sang-Hwan Hyun

**Affiliations:** 1 Laboratory of Veterinary Embryology and Biotechnology (VETEMBIO), Veterinary Medical Center and College of Veterinary Medicine, Chungbuk National University, 1 Chungdae-ro, Seowon-gu, Cheongju, 28644 Republic of Korea; 2 Institute for Stem Cell & Regenerative Medicine (ISCRM), Chungbuk National University, 1 Chungdae-ro, Seowon-gu, Cheongju, 28644 Republic of Korea; 3 Department of Neurosurgery, Chungbuk National University Hospital, Chungbuk National University, College of Medicine, Cheongju, 28644 Republic of Korea; 4 Department of Pathology, Chungbuk National University Hospital, Chungbuk National University, College of Medicine, Cheongju, 28644 Republic of Korea

**Keywords:** Atypical meningioma, Primary cancer cell line, Whole-exome sequencing, Stem cell

## Abstract

**Background:**

Meningiomas are the second most common primary tumors of the central nervous system. However, there is a paucity of data on meningioma biology due to the lack of suitable preclinical in vitro and in vivo models. In this study, we report the establishment and characterization of patient-derived, spontaneously immortalized cancer cell lines derived from World Health Organization (WHO) grade I and atypical WHO grade II meningiomas.

**Methods:**

We evaluated high-resolution 3T MRI neuroimaging findings in meningioma patients which were followed by histological analysis. RT-qPCR and immunostaining analyses were performed to determine the expression levels of meningioma-related factors. Additionally, flow cytometry and sorting assays were conducted to investigate and isolate the CD133 and CD44 positive cells from primary atypical meningioma cells. Further, we compared the gene expression profiles of meningiomas and cell lines derived from them by performing whole-exome sequencing of the blood and tumor samples from the patients, and the primary cancer cell lines established from the meningioma tumor.

**Results:**

Our results were consistent with earlier studies that reported mutations in *NF2*, *SMO*, and *AKT1* genes in atypical meningiomas, and we also observed mutations in *MYBL2*, a gene that was recently discovered. Significantly, the genomic signature was consistent between the atypical meningioma cancer cell lines and the tumor and blood samples from the patient.

**Conclusion:**

Our results lead us to conclude that established meningioma cell lines with a genomic signature identical to tumors might be a valuable tool for understanding meningioma tumor biology, and for screening therapeutic agents to treat recurrent meningiomas.

## Background

Meningiomas are the second most common primary intracranial tumors of the central nervous system, comprising nearly 30% of all primary brain tumors with annual incidence rates ranging from 1.3 to 7.8 and age-standardized mortality rate around 0.3 deaths per 100,000 individuals [1–3]. According to the World Health Organization (WHO) classification criteria, meningiomas are histologically classified into three main subtypes: benign (grade I, low-grade), atypical (grade II), and malignant meningiomas (grade III, high-grade) [4]. Surgical resection of low-grade meningiomas offers a better survival to patients; however, up to 18% of benign meningiomas, 40% of atypical meningiomas, and 80% of malignant meningioma recur within 5 years of complete excision [5–7]. Chemotherapeutic interventions have largely been unsuccessful for meningioma therapy, and refractory or recurring meningiomas are instead treated with surgery and radiotherapy [8, 9]. Here is an urgent need for novel therapeutic approaches based on effective molecular targets in order to improve long-term control of meningioma.

Lack of sufficient clinical predictive power remains one of the most critical obstacles in the development of novel study models [10]. Over recent years, patient-derived xenograft (PDX) models, which are created by grafting of patient-derived cancer cells into immunodeficient mice [11], have emerged as important tools for translational research. Efforts are underway to establish xenograft models in benign and malignant meningiomas [12, 13]. The success of xenograft models depends on reliable and biologically relevant primary cancer cell lines. Most of the well-characterized cell lines are derived from malignant meningiomas (grade III) [14–16]; however, there is a paucity of cell lines derived from grade I [17, 18] and grade II [19] meningiomas. The available atypical meningioma cell lines have been artificially-immortalized by viral transduction to induce in vivo expression of the human telomerase reverse transcriptase gene (*hTERT*), human papillomavirus E6/E7 oncogenes, or SV40 large T antigen. However, the use of these cell lines as a meningioma model comes with the caveat that it is difficult to know how artificial immortalization might impact the biology of these tumors.

Over the past decade, novel methods of high-throughput DNA sequencing, termed as next-generation sequencing (NGS), have been developed. These technologies have provided new insights into the genomic characterization of tumors, and the complex processes that occur throughout cancer progression [20–23]. In meningioma, an inactivating mutation of a tumor suppressor gene, the neurofibromatosis type 2 (*NF2*) gene, is a well-known genetic alteration [24]. *NF2* is thought to be involved in meningioma initiation rather than progression [4]. In addition, recent genomic analyses of meningioma using next-generation sequencing have identified mutations in the TNF receptor-associated factor 7 (*TRAF7*), the Kruppel-like factor 4 (*KLF4*), the v-Akt murine thymoma viral oncogene homolog 1 (*AKT1*), and the smoothened (*SMO*) gene [25, 26]. Interestingly, such mutations were found to be associated with tumorigenesis and progression of *NF2* independent meningiomas [7]. The *TRAF7* and *KLF4* are transcription factors thought to drive tumor initiation, induction of pluripotency and maintenance of stemness [27, 28]. AKT1 mutations result in downstream activation of the mTOR oncogenic pathway [29] and SMO mutations cause activation of the Hedgehog signaling pathway rendering increased proliferation of meningioma cells [30]. Despite several other genetic or chromosomal alterations having also been reported in meningioma tumors, NGS has been used in a very limited number of studies related to genomics of patient-derived atypical meningioma [25, 26], which has a poor treatment compliance and a high recurrence rate. Furthermore, although cancer cell lines have been commonly used as a suitable in vitro model for the screening and testing of cancer therapeutics [31], there has been no comprehensive studies comparing mutations in tumor-derived cell lines with those in primary tumors. This is needed to determine whether the cell lines have the same mutation blueprint as the parent meningioma tumors.

In this study, we report the establishment and comparative characterization of patient-derived, spontaneously immortalized cancer cell lines from grade I and II meningiomas. We sequenced DNA from a grade II meningioma cancer cell line using a whole-exome sequencing technique and identified somatic copy-number alterations (SCNAs), rearrangements, mutations, and insertions and/or deletions throughout the cancer-associated genes. Moreover, we compared the genomic profile of meningioma-derived cell lines to the original patient tumor to analyze their suitability as a suitable meningioma model.

## Materials and methods

### Ethics statement

Experimental procedures for this study were approved by the Ethics Committee, and permission was obtained from the institutional review board of Chungbuk National University Hospital (IRB No.: 2016-08-009-002). Written informed consents were obtained for all the patient samples.

### Chemicals

All chemicals were purchased from Sigma-Aldrich Chemical Company (St. Louis, MO, USA) unless stated otherwise.

### Isolation and primary culture of cancer cells from brain tumor tissue

Tumor samples from five human meningioma patients were surgically removed and transported to the lab in a sterile tube containing fresh Hank’s Balanced Salt Solution (HBSS, Gibco, Carlsbad, CA) at 4 °C. The primary culture of meningioma was performed as previously described [32]. Briefly, tumor tissues were cut and trypsinized for 30 min with 0.1% trypsin (Gibco) at 37 °C. Cells were seeded at a density of 5 × 10^5^ cells/ml in a 6-well plate. Cells were cultured in N2/B27 medium, which is composed of Dulbecco’s modified Eagle’s medium (DMEM)/F12 medium supplemented with 1 × B27, 0.5 × N2, 1% non-essential amino acids, 1% glutamine, 0.1 mM β-mercaptoethanol, 1% antibiotic–antimycotic (all Gibco), 10 ng/ml EGF, and 4 ng/ml FGF-2 (Invitrogen Corporation) under 3% O_2_ and 5% CO_2_. The medium was changed every alternate day and the cells were sub-cultured once they achieved 90% confluency. The early (< 3 passages) or late (> 10 passages) passage cells were used for subsequent experiments.

### Tumorsphere culture

Tumorspheres were seeded in the wells of poly-d-lysine coated plated (Sigma), using a Pasteur pipette and grown in N2/B27 medium supplemented with human recombinant FGF-2 and EGF (both at 20 ng/ml). Secondary and tertiary spheres were obtained by dissociating the primary spheres and re-plated for further characterization.

### Immunohistochemistry

Immunohistochemical staining was performed using the Immunostainer BENCHMARK XT (Ventana medical systems, Tucson, AZ, USA) according to the manufacturer’s instructions. Anti-Ki-67 (MIB1, Dako, 1:200) was used as the primary antibody. Briefly, 4 µm-thick sections of paraffin-embedded tissue were transferred onto poly-l-lysine coated adhesive slides and dried at 62 °C for 30 min. After epitope retrieval, the samples were incubated with primary antibody followed by biotinylated secondary antibody, and signals were developed using peroxidase-labeled streptavidin and 3,3′-diaminobenzidine (DAB). Slides were counterstained with Harris’ hematoxylin. Positive control samples were stained with each batch.

### Cytogenetic analysis

The confluent monolayer of the meningioma-derived primary cell line was treated with colcemid (10 µg/ml, Gibco, Carlsbad, CA, USA) for 3–5 h to induce metaphase arrest. Subsequently, karyotyping analysis was performed as previously described [33].

### Flow cytometry and cell sorting

For the analysis of cell-surface markers, a total of 500,000 cells/ml were stained with 0.5 ml fluorescence-activated cell sorting (FACS) buffer in Dulbecco’s phosphate-buffered saline (PBS) with 1% fetal bovine serum (FBS) for 30 min at 4 °C. Antibodies against CD133-APC (Biolegend, 372805, San Diego, CA, USA) and CD44-FITC (Biolegend, 338803, San Diego, CA, USA) were used for the flow cytometry analysis and sorting. Stained cells were analyzed using the SH800 Cell Sorter (Sony Biotechnology Inc., Tokyo, Japan) equipped with SH800 software.

### Gene expression analysis by qRT-PCR

The mRNA expression levels of *Vimentin*, *Nestin*, and *hTERT* were analyzed in the human meningioma cells using qRT-PCR as previously described [32], except that RN18S served as the internal control in our study. The reactions were carried out in 40 cycles with the following steps: denaturation at 95 °C for 30 s, annealing at 55 °C for 30 s, and extension at 72 °C for 30 s. The sequences of the primers used in this study are listed in Additional file 1: Table S1. The experiments were repeated three times and the relative gene expression was determined by the 2^−ΔΔCt^ method.

### Whole-exome sequencing

A total of 0.1–0.5 μg of fragmented DNA was prepared to construct libraries using the SureSelect Human All Exon Kit V5 (Agilent, Inc., USA) following the manufacturer’s protocol. Briefly, the quantified genomic DNA sample was randomly fragmented by a Covaris sonicator (Massachusetts, USA) followed by adapter ligation, purification, hybridization, and PCR. The constructed libraries were analyzed by Agilent 2100 Bioanalyzer and Illumina HiSeq 2500 (Theragen Etex Bio Institute, Suwon, Korea) according to the manufacturer’s recommendations. Raw images were processed by HCS1.4.8 for base-calling with default parameters and the sequences of each sample were generated as 101 bp paired-end reads.

### Bioinformatics analysis

Sequence reads were aligned to the human reference genome (build 37), using BWA0.7.12. Post-processing of reads for removal of PCR duplicates, merging, and indexing was performed using Picard1.92. The Genome Analysis Toolkit (GATK) was used for the recalibration of base quality, variant calling, filtration, and evaluation. Quality scores generated by the sequencer were recalibrated by analyzing the covariation among the reported quality score, position within the read, dinucleotide, and probability of a reference mismatch. Local realignment around small insertions and deletions (indels) was performed using GATK’s indel realigner to minimize the number of mismatching bases across all the reads. Statistically significant non-reference variants, single nucleotide substitutions (SNS), and small indels were identified using the GATK Unified Genotyper. The SnpEff was used to annotate the genetic variations and predict their effects.

### Sanger sequencing

Sanger sequencing to validate results from NGS was performed on the DNA Engine Tetrad 2 Peltier Thermal Cycler (BIO-RAD) using the ABI BigDye (R) Terminator v3.1 Cycle Sequencing Kit (Applied Biosystems), following the manufacturer’s protocol. Single-pass sequencing was performed on each template using primers listed in Additional file 1: Table S1. The fluorescent-labeled fragments were purified to remove the unincorporated terminators and dNTPs by following the manufacturer’s protocol. The samples were analyzed using an ABI 3730xl DNA Analyzer (Applied Biosystems).

### Statistical analysis

The data were statistically analyzed using SPSS 17.0 (SPSS, Inc., Chicago, IL, USA). The gene expression data were compared by one-way ANOVA, followed by Duncan’s or multiple range test Student’s t-test. All results are expressed as the mean ± SEM. Results with P < 0.05 were considered statistically significant.

## Results

### Neuroimaging findings and histological analysis of the original tumor

**Fig. 1:**
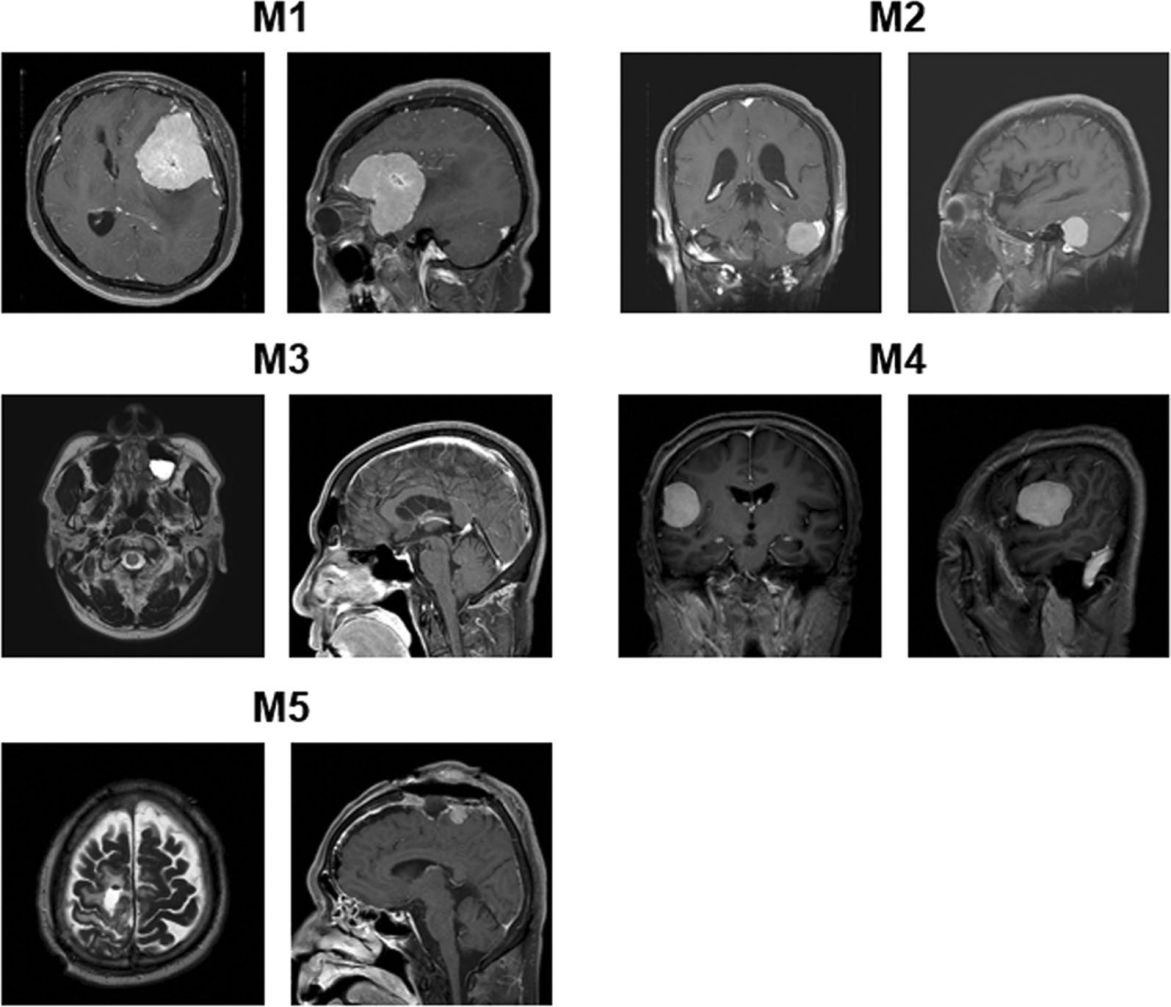
Neuroimaging of original brain tumor. M1 Preoperative T1 axial, sagittal with Gd MRI showing highly vascular extra-axial mass on left sphenoid area. Lateral sphenoid ridge meningioma that compressed the brain and caused a shift of the midline structure; M2 Preoperative T1 coronal and sagittal Gd MRI showing hypercellular mass left sigmoid-transverse sinus junction. This tumor was attached to the sinus and compressed cerebellum; M3 Preoperative T1 axial coronal Gd MRI showing right convexity meningioma. This tumor arose from the dura, and invaded right frontal lobe; M4 Preoperative T1 coronal, sagittal Gd MR showing convexity meningioma that compressed the right frontal lobe. This hyper-intense mass attached to the dura was well marginated; M5 Postoperative T2-weighted axial T1 Gd sagittal MRI showing remaining fuzzy mass that invaded and attached to the left motor cortex; this falx meningioma showed some peritumoral edema

We used high-resolution 3T MRI neuroimaging finding to evaluate the tumors in meningioma patients. As shown in Fig. 1, the focal extra-axial tumor was located in the left frontotemporal convexity of the M1 patient. The mass measured 5.4 × 5.9 cm in size and was associated with significant peritumoral edema. The hypercellular mass (max. diameter: 25 mm) was detected adjacent to the left sigmoid-transverse sinus junction compressing the cerebellum of the M2 patient. The MRI showed a dural based enhancing mass at the right frontal convexity (max. diameter: 1.7 cm) with petechial hemorrhages in both frontal lobes and right external capsule in the M3 patient. The well-defined and demarcated extra-axial mass (diameter: 4.2 × 3.2 × 2.7 cm) attached to the dura was observed in the M4 patient at the right frontotemporal convexity. Moreover, the MRI imaging demonstrated irregular, fuzzy, mass-invaded, and attached left motor cortex along the falx with peritumoral edema in the right parasagittal area of the M5 patient.

**Fig. 2:**
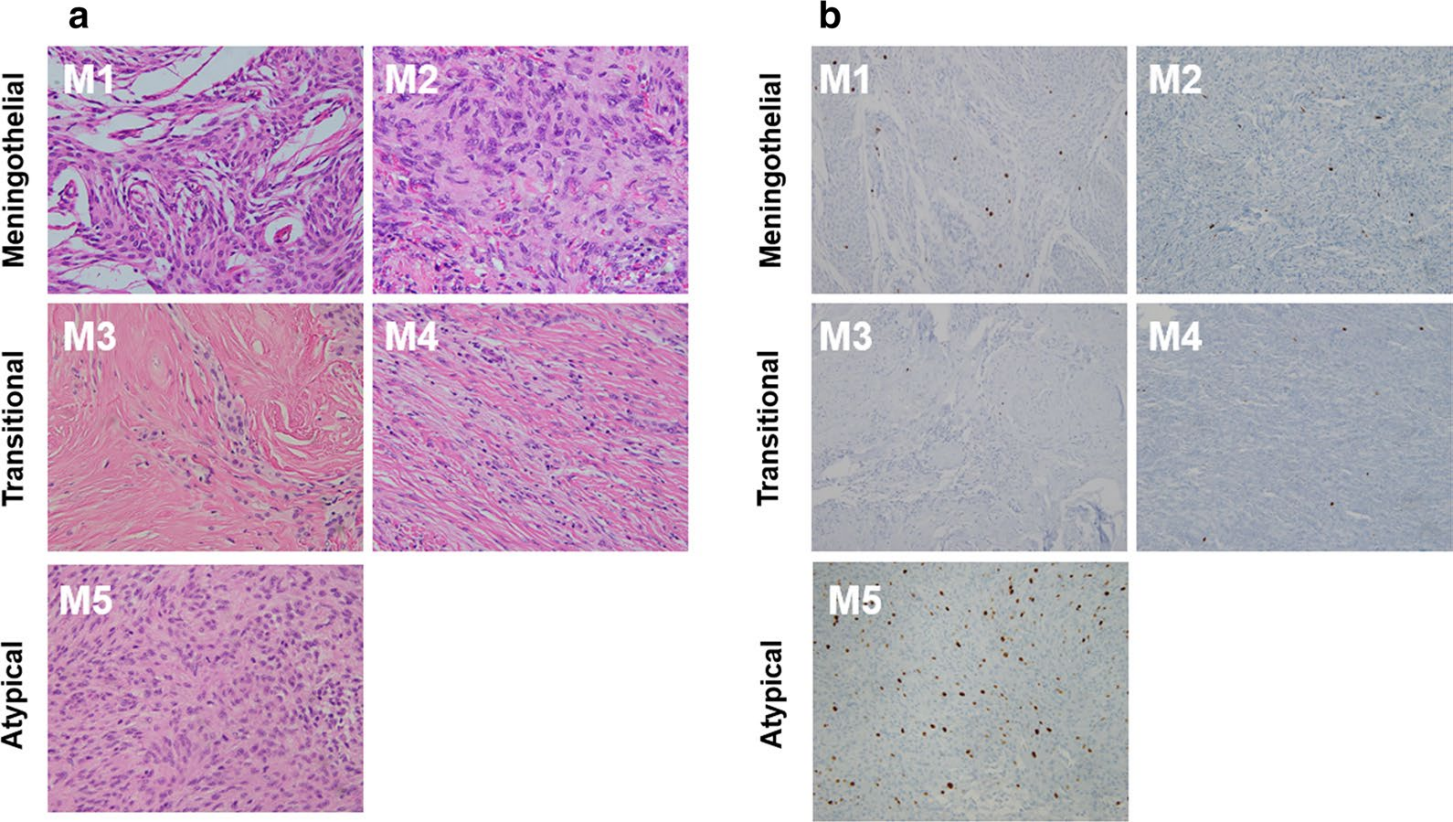
Representative histological images in human meningioma tumors. **a** Meningothelial meningioma characterized by sheets, whorls, and syncytia of neoplastic cells with vesicular nuclei (M1 and M2). Transitional meningioma showing mixed patterns with meningothelial and fibroblastic features (M3 and M4). Atypical meningioma (M5). Magnification, 400× ; hematoxylin and eosin (H&E) staining. **b** Photomicrograph showing immunohistochemical staining of proliferation index, Ki-67, in human meningothelial (M1 and M2), transitional (M3 and M4), and atypical (M5) meningiomas

Histological analysis showed that meningothelial meningioma (M1) was characterized by sheets, whorls, or syncytia of neoplastic cells with round or oval centrally located nuclei with dispersed chromatin, smooth nuclear profiles, and small indistinct nucleoli (Fig. 2a). The tumor cells of the other meningothelial meningioma (M2) had vesiculous nuclei with prominent nucleoli. The transitional meningiomas (M3 and M4) consisted of syncytial-looking tumor cells merged with spindle-shaped cells, thereby resembling the fibrous variant. Atypical meningioma (M5) exhibited more mitoses, necrosis, sheet-like growth, small cellular changes, increased cellularity, and prominent nucleoli with direct invasion into brain parenchyma compared to the other meningiomas. Although the meningothelial (M1 and M2) and transitional (M3 and M4) meningiomas showed occasional proliferating cells stained with the Ki-67 marker, the atypical meningioma (M5) showed an increased Ki-67 proliferation index with about 20% of the tumor cells having a stained nucleus compared to the other meningiomas (Fig. 2b).

### Comparative characterization of morphological and molecular features of primary cancer cell line

**Fig. 3:**
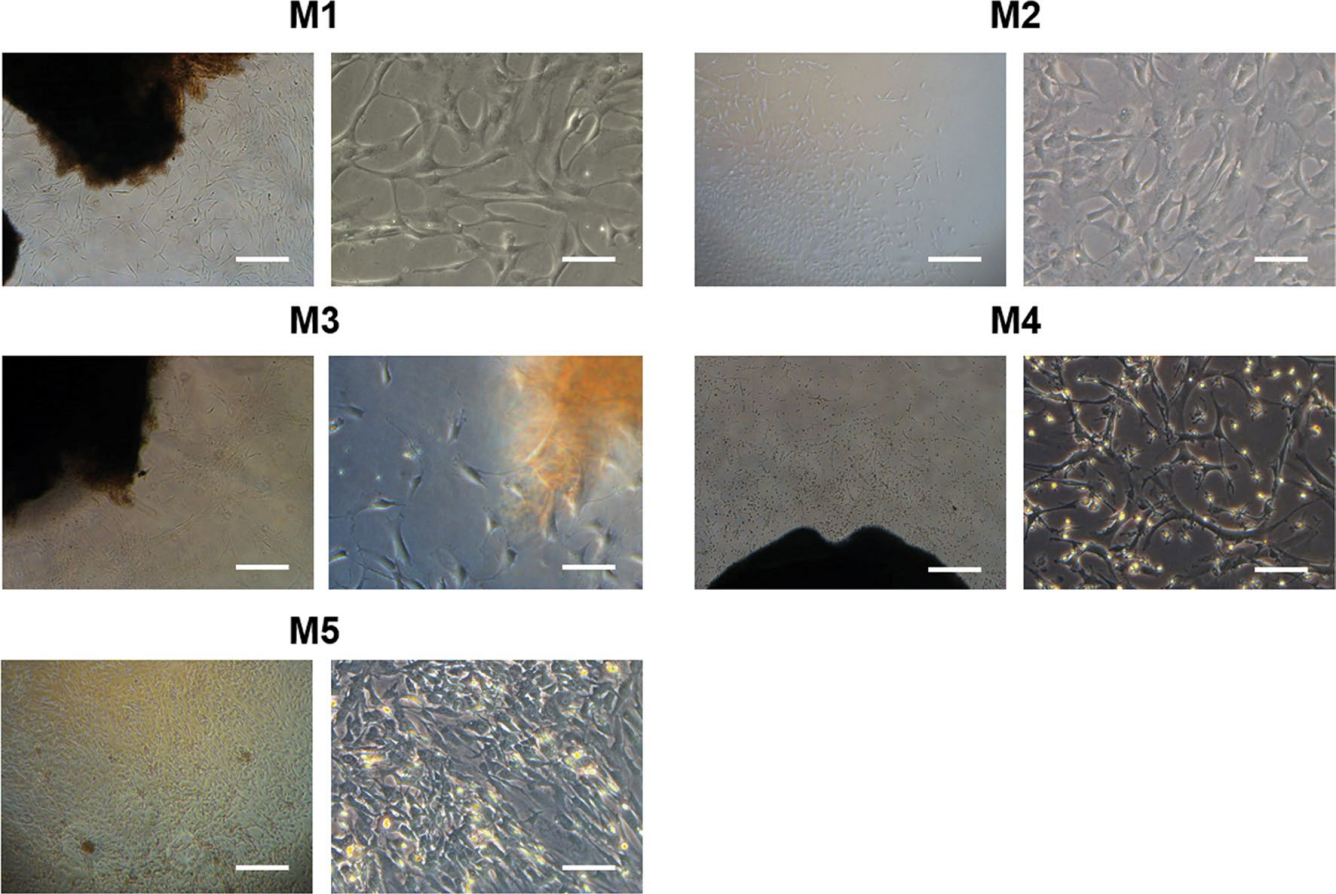
Representative phase contrast microscopy analysis of patient-derived primary brain tumor cells. The spindle–shaped cells account for the majority of the cell population in meningioma cell lines. Scale bar = 50 µm

**Fig. 4:**
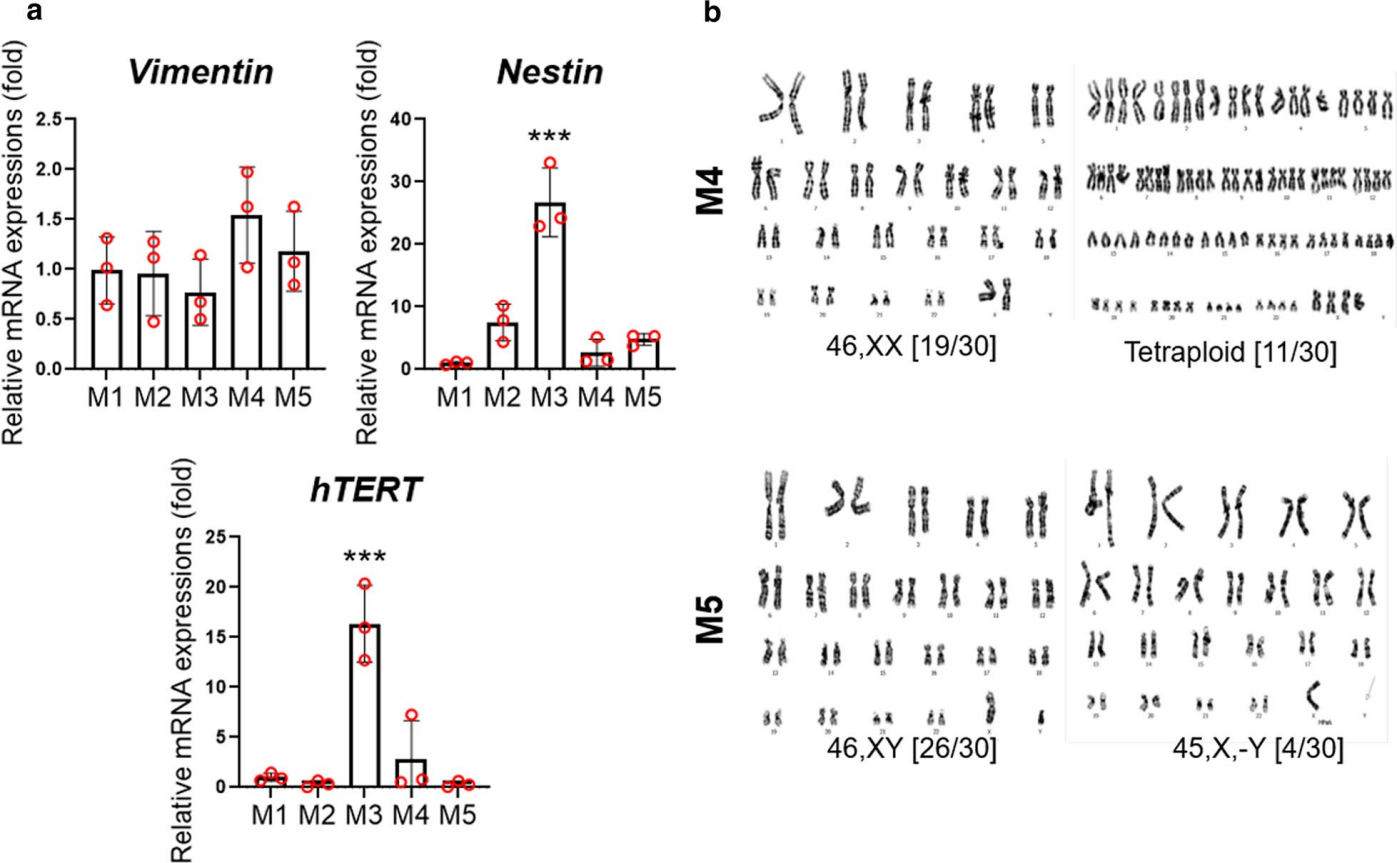
mRNA expression and cytogenetic analysis of human primary meningioma cell lines. **a** qRT–PCR analysis of *Vimentin*, *Nestin*, and *hTERT* in human primary meningioma cell lines. The value represents mean ± SEM. Data were analyzed by one-way ANOVA. Asterisks indicate statistical significance (***p < 0.001). (b) G–banded karyotype analysis showing various numerical chromosomal aberrations

**Fig. 5:**
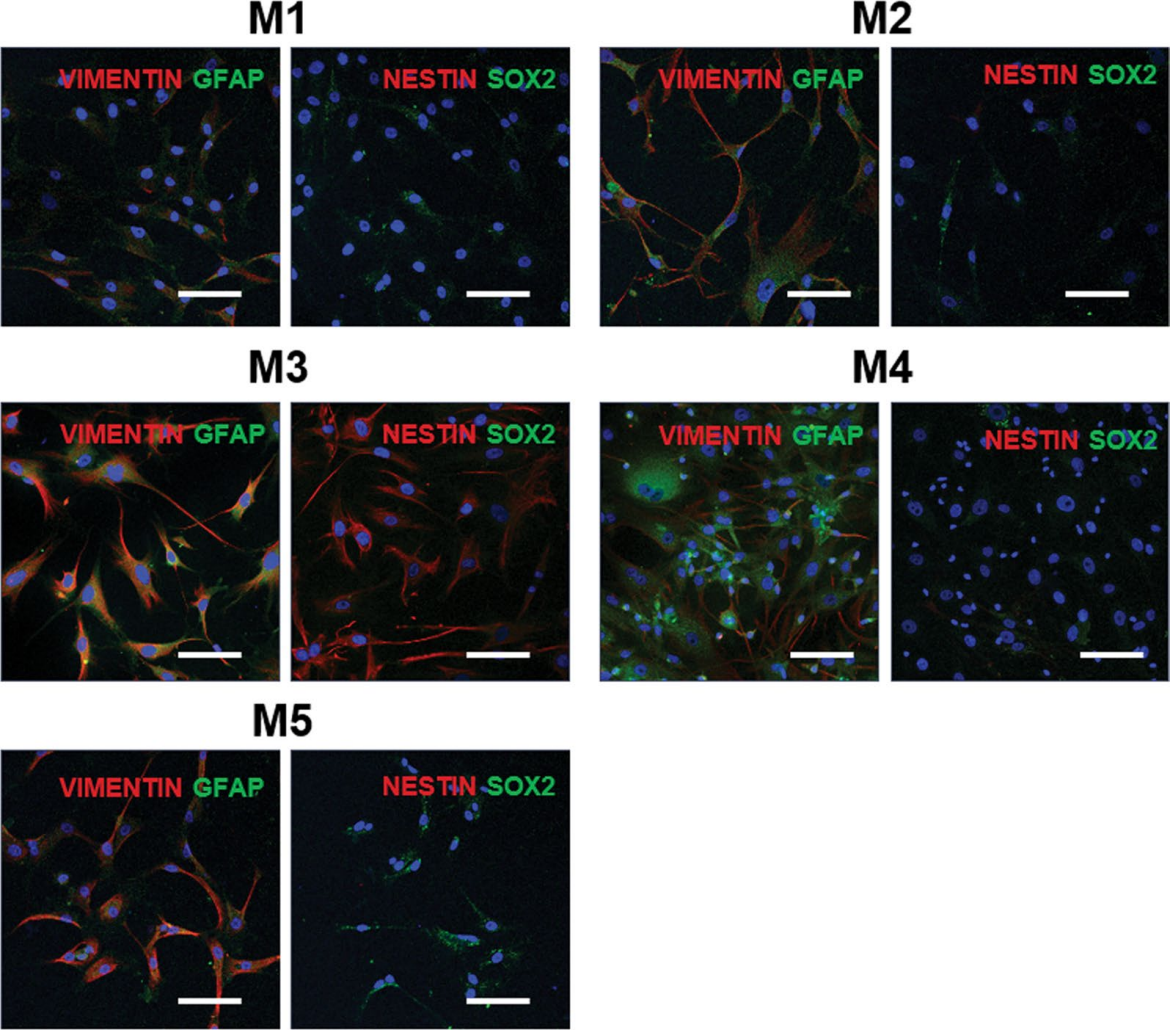
Double-immunofluorescence labeling of human primary meningioma cell lines. Red fluorescence labeling indicates NESTIN or VIMENTIN. Green fluorescence labeling indicates SOX2 or GFAP. Nuclei are counterstained with Hoechst (blue). Scale bar = 100 µm

To establish primary cancer cell lines, we performed primary cultures of meningioma tumor samples (Table 1). Almost all the primary meningioma cell lines showed homogeneously spindle-shaped cell populations with large nuclei (Fig. 3 and Additional file 2: Fig. S1). However, the M4 cell line had mixed heterogeneous cell population with spindle-shaped and rounded cells which resembled those from higher passages [34]. To perform the molecular characterization of these primary cell lines, we analyzed the mRNA expression from these cells using real-time qPCR (Fig. 4a). We first investigated whether these cells expressed meningioma markers. Our results showed that all the cancer cell lines expressed *Vimentin,* a meningioma marker, along with endogenous human telomerase catalytic subunit (hTERT). The *hTERT* mRNA expression in the M3 cell line was significantly higher than in the other cell lines. Interestingly, it also showed a significantly higher mRNA expression level of *Nestin* compared with all the meningioma cell lines. These results suggested that *hTERT* mRNA expression correlated with the *Nestin* level in our primary meningioma cell lines. Unexpectedly, the expression level of *Nestin* and *hTERT* in atypical meningioma cells was not higher than what was observed in the other benign meningioma cells. G-banding karyotypes from meningioma cell lines showed various numerical but not structural chromosomal aberrations (Fig. 4b). A near-tetraploid karyotype was found in 11 of the 30 metaphases (36.7%) seen in the transitional meningioma cell line (M4) and the loss of the Y chromosome was observed in 4 of the 30 metaphases (13.3%) obtained from an atypical meningioma cell line (M5). Using immunofluorescence analysis, the protein expression of Nestin, Sox2, Vimentin, and GFAP was analyzed in these primary cancer cell lines (Fig. 5). Our results showed a homogenous expression of Vimentin in all primary cancer cell lines, whereas a differential expression pattern of Nestin was noted. These results were consistent with the mRNA expression levels observed in these cells. Collectively, these results revealed differential characteristics from primary cancer cell lines derived from different grades of meningiomas.

**Table 1:** Information of brain tumor specimens

Cell line	Age	Sex	WHO grade	Position	Pathology
M1	56	F	I	Left sphenoid ridge	Meningothelial Meningioma
M2	59	F	I	Left posterior fossa	Meningothelial Meningioma
M3	62	M	I	Right frontal lobe	Transitional Meningioma
M4	65	F	I	Right convexity	Transitional Meningioma
M5	57	M	II	Right frontal lobe	Parasagittal Atypical Meningioma

### Characterization of atypical meningioma cancer cell line

**Fig. 6:**
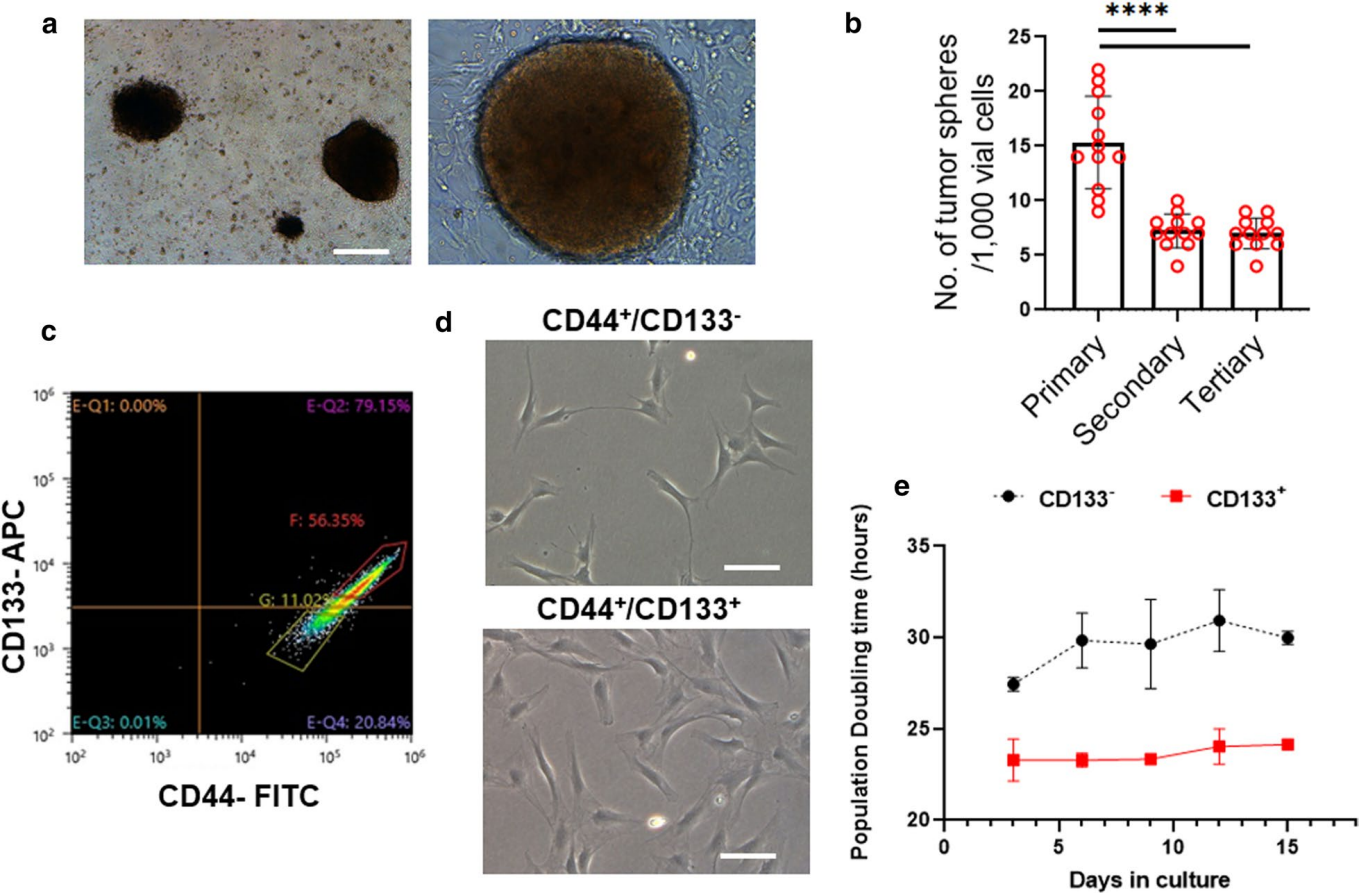
Tumorsphere formation of WHO grade II atypical meningioma cell line. **a** Representative image of primary tumorsphere from atypical meningioma cancer cell line under treatment with EGF and FGF2. Scale bars = 100 µm. **b** Number of primary, secondary, and tertiary spheres obtained from atypical meningioma cancer cell line per 10,000 viable cells plated. The number of neurospheres was determined 10 days after plating. The value represents mean ± SEM. Data were analyzed by one-way ANOVA. Asterisks indicate statistical significance (****p < 0.0001). **c** Immunophenotypic characterization by flow cytometry assay showing the expression of dual markers CD44/CD133 in primary atypical meningioma cancer cell line. **d** Representative image of sorted CD133^+^ or CD133^−^ cell population from CD44^+^ atypical meningioma cancer cell line. Scale bar = 100 µm. **e** Population doubling time of sorted CD133^+^ or CD133^−^ cell population from CD44^+^ atypical meningioma cancer cell line

To specifically analyze the characteristics of grade II meningioma, we focused on the atypical meningioma-derived primary cancer cell line. We analyzed the tumorsphere forming ability of atypical meningioma cell lines (Fig. 6a). Further, the self-renewing capacity of the tumorspheres was assayed by gently dissociating them, followed by plating and culture of the single cells obtained. We continued to passage these tumorspheres to determine whether they could form secondary, tertiary, and quaternary tumorspheres. Our results showed that the single cells derived from primary tumorspheres produced secondary tumorspheres after 7 days, indicating that the cells in tumorspheres were capable of self-renewal and proliferation. However, compared to the primary tumorsphere, the numbers of secondary and tertiary tumorspheres decreased significantly (Fig. 6b). Our results from FACS showed the expression pattern of the cancer stem cell markers CD44/CD133 [35] in an atypical meningioma cell line (Fig. 6c). The cytometry dot plot of CD133 expression in atypical meningioma cells showed that 79.15% of the cells were CD133^+^ (gate Q2), while 20.84% of the cells were CD133^−^ (gate Q4). The sorted double-positive subpopulations (CD44^+^/CD133^+^) derived from the atypical meningioma cell line (Fig. 6d) showed a significantly shorter population doubling (PD) time (Fig. 6e) compared to the CD44^+^CD133^−^ populations, suggesting extensive proliferation and stem-like properties of the CD133^+^ cells.

### Sequencing analysis of an atypical meningioma cancer cell line

**Fig. 7:**
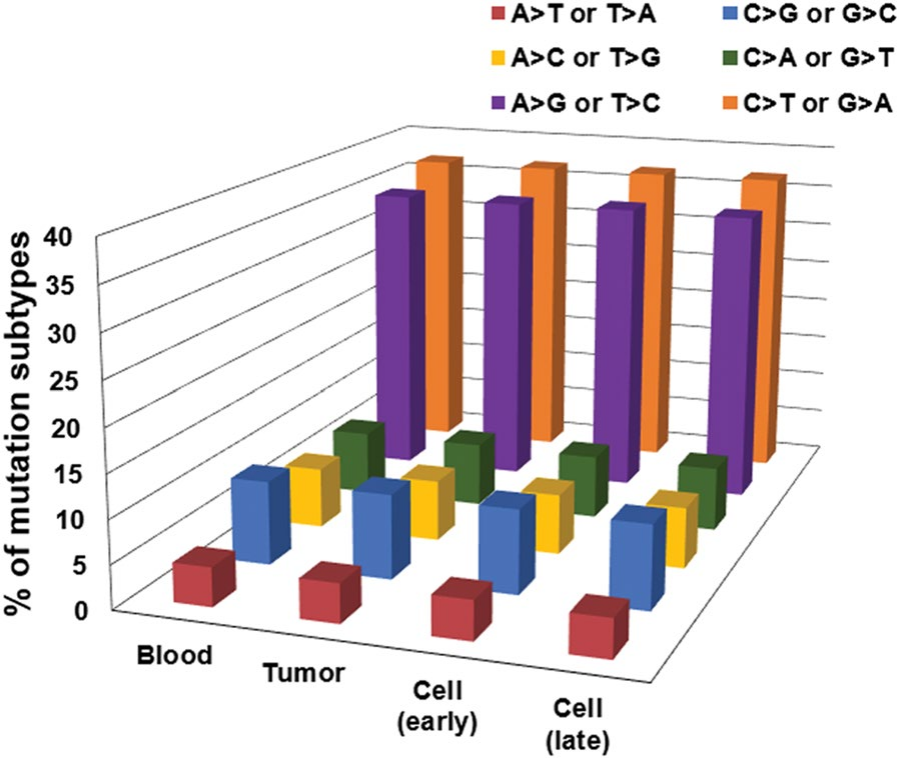
Qualified graph of base transversions across all four samples from atypical meningioma. Base substitutions were divided into six categories to represent the six possible base changes (each category is represented by a different color)

To define the genomic characteristics of the established atypical meningioma cell line, we performed whole-exome sequencing on DNA extracted from the blood and tumor samples from the atypical meningioma patient, and the tumor-derived primary cell lines at the early and late passage. A total of 184,696 single nucleotide variants (SNVs) were identified through whole-genome sequencing of the meningioma sample, including 12,057 inserted and deleted sequences (indels) and 172,639 single nucleotide polymorphisms (SNPs) (Additional file 3: Fig. S2a). Further, chromosomes 1, 6, 12, and 10 showed more SNVs (Additional file 3: Fig. 2Sb). All groups showed similar mutations in studied genes (Additional file 3: Fig. S2c, d). The most frequent SNPs and indels were intronic, exonic, and downstream or upstream of genes with silent or missense mutations. To assess the quality of SNPs, we computed the transition-to-transversion (TS/TV) ratio/sample as an important parameter of possible random sequence errors. All the TS/TV ratios were similar (> 2.51) to each other (Additional file 1: Table S2). Of these SNVs, a high prevalence of C > T base (G > A base in the complementary strand) transversions (36.82%) were observed in the tumor sample (Fig. 7, Additional file 1: Table S3).

**Fig. 8:**
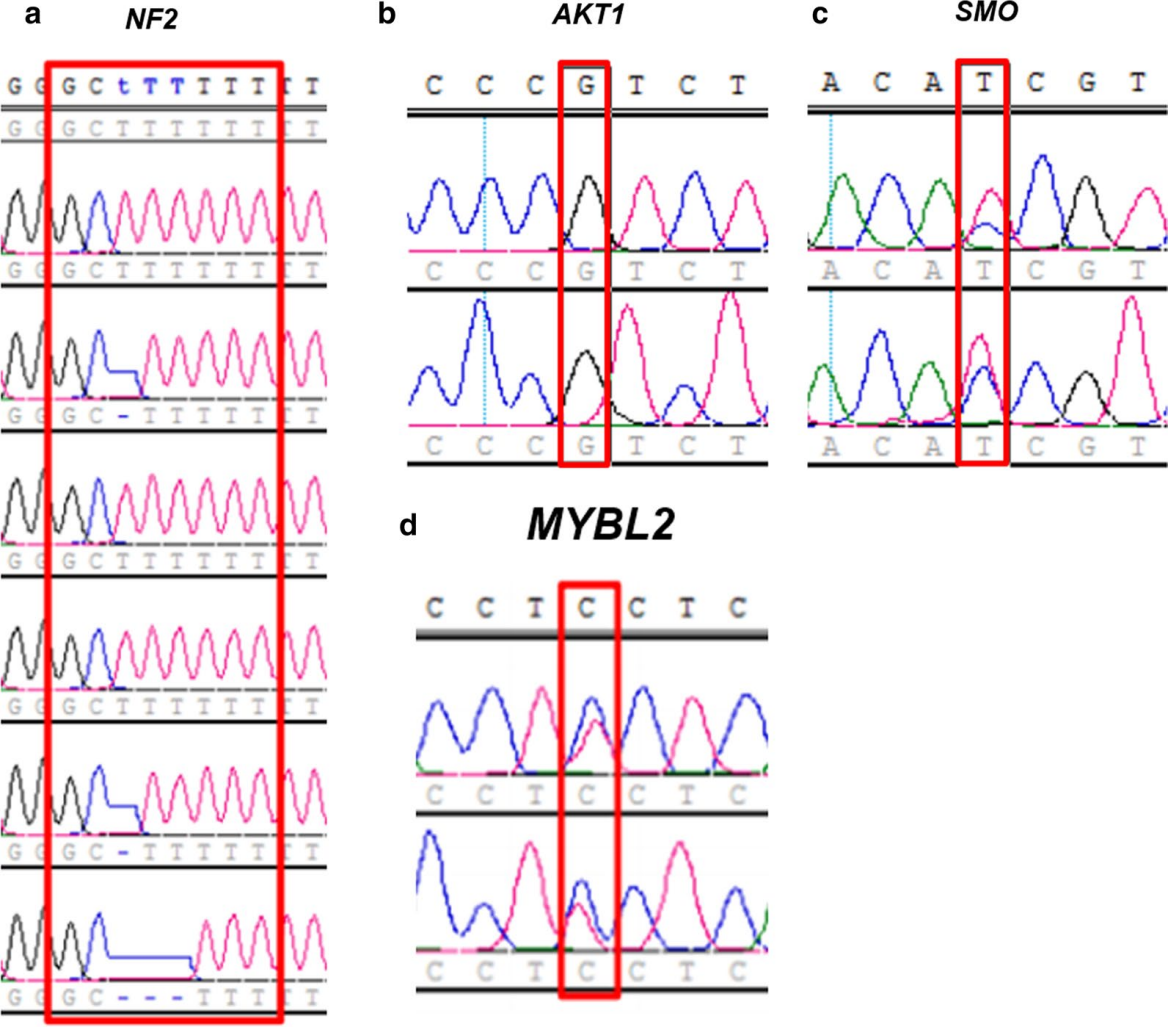
Sanger sequencing of representative genes from atypical meningioma identified in this study. Sanger sequencing of *NF2*, *AKT1*, *SMO*, and *MYBL2* genes from atypical meningiomas analyzed in this study. Red box area indicates nucleotide substitutions: chr.22CTTT > C/CT/CTT in *NF2* (**a**), chr.14A > G in *AKT1* (**b**), chr.7C > C/T in *SMO* (**c**), and chr.20C > C/T in *MYBL2* (**d**)

In the present study, all the sample groups had mutations in the meningioma-driver genes such as *NF2*, *AKT1*, *SMO*, *TRAF7*, and *KLF4* (Table 2). Although the early cell lines showed genotypes similar to the parent tumors, the late cell lines showed some differences in the *NF2* genotype. Subsequently, to confirm the whole-exome sequencing results, we conducted Sanger sequencing of the PCR amplicons to validate these mutations on all exons and exon–intron boundaries of *NF2*, *AKT1*, and *SMO* and confirmed that these variants were genuine (Fig. 8). Moreover, we confirmed the mutated status of the newly identified *MYBL2* (V-Myb avian myeloblastosis viral oncogene homolog-like 2) gene in our patient tumor sample (Table 3). Results from our study suggest that the established primary cancer cell lines recapitulated the genomic mutations observed in tumor samples derived from the atypical meningioma patient.

**Table 2:** Summary of SNVs observed in *NF2*, *TRAF7*, *SMO*, AKT1, and *KLF4* genes

Chromosome	Gene	Position	Vartype/Effect	Genotype				Variant	Biotype	EXAC_AC	EXAC_AN
				Blood	Tumor	Early	Late				
chr22	NF2	30038152	SNP/intron_variant	0/1	0/1	0/1	0/1	c.364-39A > C	Protein_coding	22478	121410
			SNP/intron_variant	0/1	0/1	0/1	0/1	c.115-39A > C	Protein_coding	22,478	121410
			SNP/intron_variant	0/1	0/1	0/1	0/1	c.238-39A > C,	Protein_coding	22,478	121410
			SNP/intron_variant	0/1	0/1	0/1	0/1	c.241-39A > C	Protein_coding	22,478	121410
			DEL/intron_variant	0/1	0/1	0/1	0/2	c.599 + 56delT	Protein_coding	.	.
			DEL/intron_variant	0/1	0/1	0/1	0/2	c.350 + 56delT	Protein_coding	.	.
			DEL/intron_variant	0/1	0/1	0/1	0/2	c.473 + 56delT	Protein_coding	.	.
			DEL/intron_variant	0/1	0/1	0/1	0/2	c.476 + 56delT	Protein_coding	.	.
			DEL/intron_variant	0/1	0/1	0/1	0/2	c.447 + 13447delT	Protein_coding	.	.
chr16	TRAF7	2201270	SNP/upstream_gene_variant	0/1	0/1	0/1	0/1	c.-12652G > C	Protein_coding	.	.
		2203456	SNP/upstream_gene_variant	1/1	1/1	1/1	1/1	c.-10466T > C	Protein_coding	120,133	121,104
		2223864	SNP/intron_variant	0/1	0/1	0/1	0/1	c.1135 + 27G > C	Protein_coding	15,920	117,352
		2223868	SNP/intron_variant	0/1	0/1	0/1	0/1	c.1135 + 31A > C	Protein_coding	15,557	117,050
		2223872	SNP/intron_variant	0/1	0/1	0/1	0/1	c.1135 + 35T > C	Protein_coding	.	120,252
chr14	AKT1	105258893	SNP/intron_variant	1/1	1/1	1/1	1/1	c.46 + 42T > C	Protein_coding	99,675	121,090
chr9	KLF4	110249505	SNP/intron_variant	0/1	0/1	0/1	0/1	c.1100-32G > A	Protein_coding (p.Gly390Gly)	21,511	121,396
			SNP/synonymous_variant	0/1	0/1	0/1	0/1	c.1170G > A	Protein_coding (p.Gly390Gly)	21,511	121,396
chr7	SMO	128845018	SNP/intron_variant	0/1	0/1	0/1	0/1	c.538-26C > T	Protein_coding	96,595	121,412
		128846328	SNP/synonymous_variant	0/1	0/1	0/1	0/1	c.1164G > C	Protein_coding (p.Gly388Gly)	96,810	121,412

**Table 3:** Identification of novel mutations in the patient tumor by using whole-exome sequencing

Chromosome	Gene	Position	Class	Variants	Biotype
chr3	SFMBT1	52966180	Predicted intracellular proteins	c.598G > T	Protein_coding (p.Glu200*)
chr3	ETV5	185797800	cancer-related genes, predicted intracellular proteins, transcription factors	c.456A > C	Protein_coding (p.Pro152Pro)
chr4	CORIN	47644083	Disease-related genes, enzymes, plasma proteins, potential drug targets, predicted intracellular proteins	c.*832G > T,c.2069-17G > T,c.1757-17G > T	Protein_coding
chr4	FSTL5	162697050	Predicted secreted proteins	c.586G > A,c.583G > A,c.583G > A	Protein_coding (p.Asp196Asn,p.Asp195Asn,p.Asp195Asn)
chr6	SNRNP48	7609079	Predicted intracellular proteins	c.993T > G	Protein_coding (p.Ser331Arg)
chr9	ADAMTSL2	136405721	Disease-related genes, plasma proteins, predicted intracellular proteins, predicted secreted proteins	c.414T > A,c.414T > A	Protein_coding (p.Asp138Glu,p.Asp138Glu)
chr10	SFMBT2	7423901	Predicted intracellular proteins	c.-41C > T,c.-41C > T,c.-41C > T,c.-41C > T	Protein_coding
chr11	MRGPRX2	19077162	G-protein coupled receptors, predicted membrane proteins	c.788T > C,c.788T > C	Protein_coding (p.Val263Ala,p.Val263Ala)
chr12	ARHGEF25	58006730	Predicted intracellular proteins	c.232G > T,c.115G > T,n.698 + 51G > T	Protein_coding (p.Gly78Cys,p.Gly39Cys)
chr12	DTX3	58006730	Predicted intracellular proteins	c.*3795G > T,c.*3795G > T,c.*3795G > T	Protein_coding
chr12	SRRM4	119594473	Predicted intracellular proteins	c.1706G > A	Protein_coding (p.Ser569Asn)
chr12	HCAR1	123214716	G-protein coupled receptors, predicted membrane proteins, transporters	c.171G > A	Protein_coding (p.Leu57Leu)
chr15	RGMA	93595562	Predicted intracellular proteins, predicted secreted proteins	c.330C > A,c.258C > A,c.258C > A,c.258C > A,c.258C > A,c.306C > A	Protein_coding (p.Ala110Ala,p.Ala86Ala,p.Ala86Ala, p.Ala86Ala,p.Ala86Ala,p.Ala102Ala)
chr17	TMEM132E	32964719	Disease-related genes, predicted membrane proteins	c.2693T > G	Protein_coding (p.Phe898Cys)
chr20	MYBL2	42328630	Cancer-related genes, predicted intracellular proteins, transcription factors	c.897C > T,c.825C > T	Protein_coding (p.Leu299Leu,p.Leu275Leu)

## Discussion

We report the establishment of in vitro primary cell lines derived from meningioma tumor samples (WHO grade I and II), and their comprehensive characterization by gene expression profiling, and a comparison between the blood and tumor samples from the patient using whole-exome sequencing.

Given the importance of the personalized prediction of tumor response and cancer progression, there is a need to develop reliable, and more importantly, patient-derived in vitro models. These models will not only be helpful in understanding the biology and pathology of meningioma but also will facilitate the development of novel therapeutic approaches in the clinic [36]. Primary cancer cell lines derived from human tumors play a critical role as an experimental model in investigating cancer biology and molecular pharmacology. The viral transduction of cells to express *hTERT* has been the most common method to immortalize primary cancer cell lines. The expression level of endogenous *hTERT* is shown to directly correlate with the degree of malignancy in cancer cells [37], and expression of *hTERT* is observed in 30–50% of all benign and nearly 100% of high-grade meningiomas [38, 39]. Despite being established without any artificial immortalization, all primary cell lines in this study showed endogenous expression of *hTERT* and a marked capacity for active proliferation, consistent with the previous report of a spontaneously immortal low-grade meningioma cell line [34]. The striking chromosomal abnormalities in these primary meningioma cell lines might be related to *hTERT* expression and telomerase activity [40]. In this study, all primary cell lines derived from benign and atypical meningioma retained the strong expression of the meningioma marker Vimentin, which has been explicitly related to tumor malignancy [4]. On the contrary, expression of Nestin and hTERT varied across cell lines, although both showed significantly strong expression in the transitional meningioma cell line M3. The expression of *Nestin* and *hTERT* has been reported to be closely correlated to progression and recurrence in meningioma [41, 42]. Therefore, the higher expression level of *Nestin* and *hTERT* in M3 cell lines might suggest a higher probability of recurrence after surgery. However, further studies are required at the protein level in meningiomas to determine whether to use these genes as recurrence or progression biomarkers.

A recent study reported that the cells grown in the presence of defined mitotic factors such as EGF and FGF2 more closely mirror the phenotype and genotype of primary tumors than do serum-cultured cell lines [43]. Therefore, in this study, we replaced serum with these mitotic factors for the culture of the established primary meningioma cell lines. Among the three pathological grades of meningiomas, grade II atypical meningioma showed increased mitotic activity as well as high cellularity of small cells with a higher nuclear to cytoplasmic ratio, and sheet-like growth as reported in previous studies [44]. Interestingly, only atypical meningioma-derived cell lines have been reported to form proliferative tumorspheres, which enabled a variety of in vitro cell-based assays in meningioma study [45]. The primary cell lines derived from atypical meningioma in our study also possessed the ability of self-renewal and proliferation. We believe that the primary cell line established in our study is suitable for studies seeking to answer patient-specific research questions as well as for screening effective novel therapeutic agents. Further, we performed FACS analysis to analyze the expression of CD44 and CD133, which are known cancer stem cell (CSC) markers and have been proposed as candidate meningioma CSCs markers [46, 47]. The atypical meningioma-derived cell line showed enhanced expression of CD44 in all the cells including CD133^+^ and CD133^−^ cell subpopulations. The sorted CD133^+^ subpopulation of meningioma cells showed enhanced proliferation compared with the CD133^−^ subpopulation and these observations were consistent with an earlier report [48]. Moreover, these CSC-related features might play an important role in recurrence of meningioma [49]. Although the CD44^+^/CD133^+^ dual-positive phenotype may imply a stem-like feature of the model, further validation studies are necessary to determine whether our atypical meningioma-derived primary cell lines meet the criteria of CSCs and are suitable for studies on cancer stem cell biology.

Although cancer cell lines provide representative genetic information for primary tumors in many cancer types [50], the results might not be completely devoid of misinterpretations due to the artifacts introduced by selection in vitro. As a result, huge differences are expected to exist between the genomes of the patient tumors and cancer cell lines derived from them [51]. Therefore, selecting a cell line with a genetic profile closer to the parent tumor is helpful in generating a relevant in vitro preclinical model. With emerging genome-wide, high throughput, highly informative big data technologies such as NGS, it is now possible to make large-scale comparisons of gene-expression profiles to characterize both the similarity and the differences between cell lines and the original tumors. Since the genomic profiles of atypical grade II meningiomas are poorly understood compared to grade I or III meningiomas, the present study has tried to fill the lacunae by focusing on the atypical meningiomas-derived primary cell lines and analyzing the gene mutations, structural and copy number changes, and mRNA expression profile of these cells. The mutation in the *NF2* gene is the most common genetic cause of meningioma. Merlin (Moesin–ezrin–radixin-like protein) is a product of the *NF2* gene and a member of the protein 4.1 family that links actin to plasma membrane proteins [52]. Several studies have reported that Merlin can function as a tumor suppressor in diverse cell types [53, 54], while mutations in *NF2* are found in approximately 60% of sporadic meningiomas [55, 56]. Our results showed that the primary atypical meningioma has an *NF2* deletion mutation in the tumor sample as well as in the primary cancer cell lines. Several other studies based on whole-genome sequencing have reported the presence of somatic mutations in the G-protein-coupled receptor, Smoothened (*SMO*), and in the mTOR pathway associated serine-threonine protein kinase, and V-AKT murine thymoma viral oncogene homologue 1 (*AKT1*) in non-*NF2* meningiomas [25, 26]. We did observe meningioma-related mutations in oncogenic *AKT1, SMO*, *TRAF7*, and *NF2* gene. Grade III meningioma are less likely to have *TRAF7*, *AKT*1, or *SMO* mutations, and exhibit genomic instability in the form of increased copy number variation [57]. Moreover, our results from exome-sequencing and their validation by Sanger sequencing revealed a novel mutation in the *MYBL2* gene, which is a transcription factor of the MYB proto-oncogene family and a critical regulator of cell proliferation, cell survival and differentiation [58]. Recently, it has been reported that *MYBL2* is a novel candidate biomarker gene for various cancer cells such as colorectal [59], gallbladder [60] and cervical cancer [61]. In meningioma, the alterations of the *MYBL2* might be involved in cancer initiation and progression by affecting cell cycle progression, resistance to therapy and favoring metastatic spread [62]. In this study, the presence of *AKT1* and *SMO* mutations along with mutated *MYBL2*, both in the *NF2*-mutant primary atypical meningioma tumor and the derived cell line, proved that the meningioma-derived primary cell line had retained the genetic signature of the original tumor. Therefore, our current work is distinct from the previous studies [25, 26, 63] in that we proved the representativeness of a meningioma-derived cell lines as an in vitro model for the preclinical cancer studies.

Long-term cultures of tumor cells with stem cell-like properties are appropriate in vitro models to develop targeted therapeutic strategies for cancer treatment [64, 65]. A limitation of this study is that there were some genetic changes in the mutations in the primary meningioma cells cultured for a long time and thus, it cannot be ruled out that these changes induced by the long-term culture may compromise the reproducibility and thereby the credibility of the research data. To overcome this limitation, it is important to develop culture conditions that facilitate the retention of the native genetic signature and properties of tumor cells; the establishment of induced pluripotent stem cells from meningioma patients may also resolve this problem. Another limitation of this study included the limited sample size of atypical meningioma. Although conducting a further study with a larger sample might improve results of sequenced samples, the current study provides preliminary insights into the translational studies of meningioma tumor biology and progression.

## Conclusion

The spontaneously immortal, patient tumor-derived meningioma cell lines established in this study could be utilized to generate xenograft tumor models and might provide a powerful tool to improve the understanding of the underlying pathobiology of meningiomas. These cell lines could be valuable tools to test novel therapeutic approaches for meningioma treatment. Moreover, the comprehensive genomic analyses of primary atypical meningioma cell lines and their comparison with the original tumor demonstrates the contribution of whole genome sequencing towards the possibility of generating reliable research models useful in developing personalized therapeutic approaches for the treatment of recurrent atypical meningioma.

## Supplementary information

**Additional file 1: Table S1.** Primers used for the gene expression and Sanger sequencing analysis. **Table S2.** Transition-to-transversion and homozygous-to-heterozygous ratios for the single nucleotide polymorphism (SNP) datasets. **Table S3.** Summary of base transversions across all four samples of atypical meningioma.

**Additional file 2: Fig. S1**. Representative phase contrast microscopy analysis of patient-derived primary brain tumor cells at late passage. Scale bar = 50 µm.

**Additional file 3: Fig. S2.** Whole exome sequencing of atypical meningioma samples including blood, original tumor, and early and late cell lines (a) Variant type in primary cancer cells derived from atypical meningioma (M5). SNP: single-nucleotide polymorphism, INS: insertion mutation, which is the addition of one or more nucleotide base pairs into a DNA sequence, DEL: deletion mutation, in which a part of a chromosome or sequence of DNA is missing. (b) The percentage of single nucleotide variants (SNVs) occurring on each chromosome. Chr, chromosome. (c) Distribution of SNP and indels in blood, tumor, and established cell lines derived from atypical meningioma. Downstream: downstream of a gene (default length: 5 K bases), Exon: variant hits a gene, Intron: variant hits an intron; technically, this indicates that it hits no exon in the transcript, Upstream: Upstream of a gene (default length: 5 K bases), (d) Graph displaying the percentage of mutation types, including silent, missense, and nonsense mutations.

## Data Availability

The datasets during and/or analysed during the current study available from the corresponding author on reasonable request.

## Abbreviations

WHO: World Health Organization
PDX: Patient-derived xenograft
hTERT: Human telomerase reverse transcriptase
NGS: Next-generation sequencing
SCNAs: Somatic copy-number alterations
HBSS: Hank’s Balanced Salt Solution
DMEM: Dulbecco’s modified Eagle’s medium
DAB: Diaminobenzidine
FACS: Fluorescence-activated cell sorting
PBS: Phosphate-buffered saline
FBS: Fetal bovine serum
qRT-PCR: Quantitative real-time polymerase chain reaction
GATK: Genome Analysis Toolkit
SNS: Single nucleotide substitutions
SNV: Single nucleotide variants
SNPs: Single nucleotide polymorphisms
TS/TV: Transition-to-transversion
CSC: Cancer stem cell
NF2: Neurofibromatosis type 2
ANOVA: One-way analysis of variance
