# Supplementary material for: Characterization and comparison of genomic profiles between primary cancer cell lines and parent atypical meningioma tumors
Source: Cancer Cell Int. 2020 Jul 28;20:345. doi: 10.1186/s12935-020-01438-x (PMC7388534; doi:10.1186/s12935-020-01438-x)
Supplement: Supplementary file 1 — Additional file 1: Table S1. Primers used for the gene expression and Sanger sequencing analysis. Table S2. Transition-to-transversion and homozygous-to-heterozygous ratios for the single nucleotide polymorphism (SNP) datasets. Table S3. Summary of base transversions across all four samples of atypical meningioma. [file 12935_2020_1438_MOESM1_ESM.docx]

**Supplementary Table 1. Primers used for the gene expression and Sanger sequencing analysis.**

| Genes | Primer sequences | Products |
| --- | --- | --- |
|  |  | size (bp) |
| *Vimentin* | 5’-GAGAACTTTGCCGTTGAAGC-3’ | 144 |
|  | 5’-CTCAATGTCAAGGGCCATCT-3’ |  |
| *Nestin* | 5’-GCCCTGACCACTCCAGTTTA-3’ | 201 |
|  | 5’-GGGAGTCCTGGATTTCCTTC-3’ |  |
| *hTERT* | 5’-GTGACCGTGGTTTCTGTGTG-3’ | 201 |
|  | 5’-AGAGGAAGTGCTTGGTCTCG-3’ |  |
| *NF2* | 5’-ATGACTCCGGAAATGTGGGA-3’ | 294 |
|  | 5’-ATCTTGACAGGCAGTACACAT-3’ |  |
| *SMO* | 5’-TGAGGTACAGAGAGGCCAAA-3’ | 328 |
|  | 5’-CGATGTAGCTGTGCATGTCC-3’ |  |
| *AKT* | 5’-CAGGAGAGAGGCTGGCAG-3’ | 367 |
|  | 5’-CTGAGAGGAGCGCGTGAG-3’ |  |
| *MYBL2* | 5’-AGCCCATCGGTACAGATCTG-3’ | 291 |
|  | 5’-GTTAGCAGGGCGATGGAATG-3’ |  |
| *TRAF7* | 5’-ATGTTAAATGTGAGCGGGCG-3’ | 456 |
|  | 5’-AGTAGACGCAGAGACACCAC-3’ |  |
| *RN18S* | 5’-CGGCTACCACATCCAAGGAA-3’  5’-GCTGGAATTACCGCGGCT-3’ | 186 |

**Supplementary Table 2. Transition-to-transversion and homozygous-to-heterozygous ratios for the single nucleotide polymorphism (SNP) datasets**

| Category | Ts | Tv | Ts/Tv | Hetero | Homo | Hetero/Homo |
| --- | --- | --- | --- | --- | --- | --- |
| Blood | 43701 | 17312 | 2.52 | 27037 | 19154 | 1.41 |
| Tumor | 43742 | 17414 | 2.51 | 27529 | 19029 | 1.45 |
| Cell (early) | 43411 | 17148 | 2.53 | 26304 | 19243 | 1.37 |
| Cell (late) | 43613 | 17287 | 2.52 | 27016 | 19054 | 1.42 |

* Ts: Number of transition, Tv: Number of transversion, Ts/Tv: ratio of the number of transitions to the number of transversions for a pair of sequences, Hetero: Number of hetero variants, Homo: Number of homo variants, Hetero/Homo: ratio of the number of hetero variants to the number of homo variants

**Supplementary Table 3. Summary of base transversions across all four samples of atypical meningioma.**

|  | A>T or T>A* | C>G or G>C | A>C or T>G | C>A or G>T | A>G or T>C | C>T or G>A |
| --- | --- | --- | --- | --- | --- | --- |
| Blood | 4.47% | 9.65% | 6.98% | 7.43% | 34.60% | 36.87% |
| Tumor | 4.49% | 9.72% | 7.03% | 7.43% | 34.51% | 36.82% |
| Cell (early passage) | 4.50% | 9.67% | 6.95% | 7.37% | 34.66% | 36.85% |
| Cell (late passage) | 4.44% | 9.66% | 7.02% | 7.42% | 34.55% | 36.91% |

*Base transversions occur both in sense and antisense strands. If A>T mutation occur in sense strand, antisense strand will harbor a T>A mutation.
