# Supplementary figures and images for: Characterization and comparison of genomic profiles between primary cancer cell lines and parent atypical meningioma tumors
Source: Cancer Cell Int. 2020 Jul 28;20:345. doi: 10.1186/s12935-020-01438-x (PMC7388534; doi:10.1186/s12935-020-01438-x)

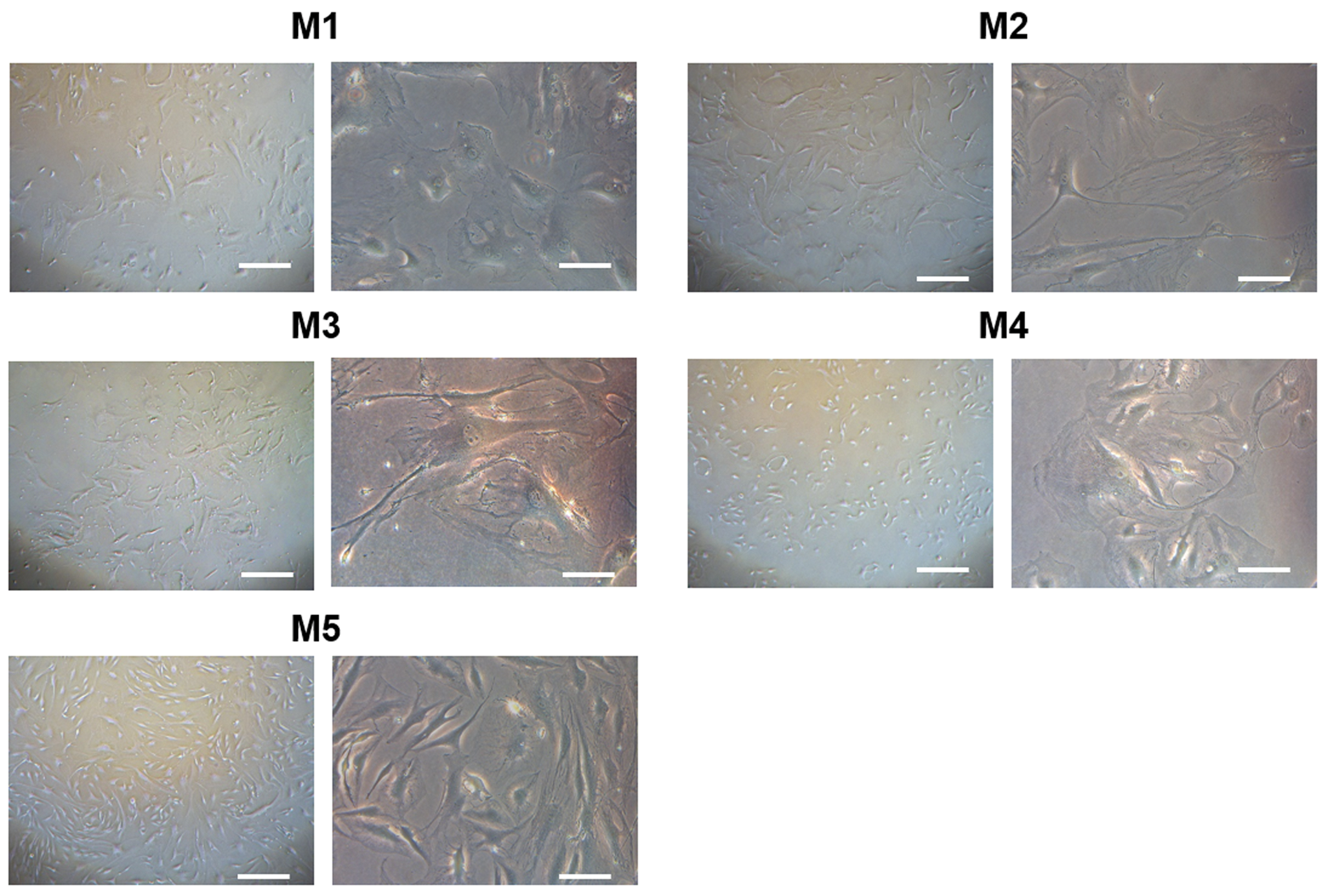

Supplement: Supplementary file 2 — Additional file 2: Fig. S1. Representative phase contrast microscopy analysis of patient-derived primary brain tumor cells at late passage. Scale bar = 50 µm. [file 12935_2020_1438_MOESM2_ESM.tif]

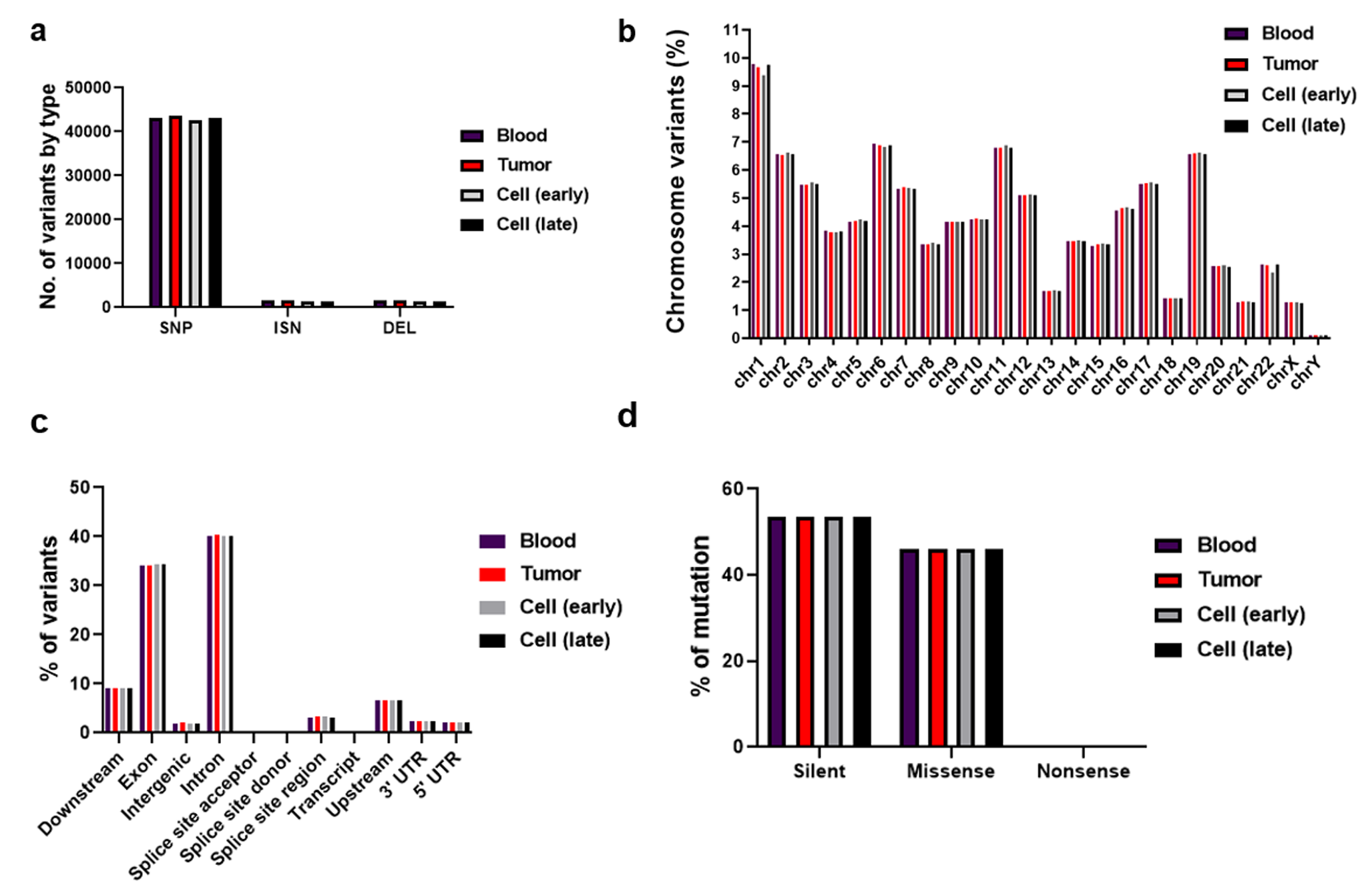

Supplement: Supplementary file 3 — Additional file 3: Fig. S2. Whole exome sequencing of atypical meningioma samples including blood, original tumor, and early and late cell lines (a) Variant type in primary cancer cells derived from atypical meningioma (M5). SNP: single-nucleotide polymorphism, INS: insertion mutation, which is the addition of one or more nucleotide base pairs into a DNA sequence, DEL: deletion mutation, in which a part of a chromosome or sequence of DNA is missing. (b) The percentage of single nucleotide variants (SNVs) occurring on each chromosome. Chr, chromosome. (c) Distribution of SNP and indels in blood, tumor, and established cell lines derived from atypical meningioma. Downstream: downstream of a gene (default length: 5 K bases), Exon: variant hits a gene, Intron: variant hits an intron; technically, this indicates that it hits no exon in the transcript, Upstream: Upstream of a gene (default length: 5 K bases), (d) Graph displaying the percentage of mutation types, including silent, missense, and nonsense mutations. [file 12935_2020_1438_MOESM3_ESM.tif]
